# Fatigue, depression, and physical impairment in multiple sclerosis

**Published:** 2014-04-03

**Authors:** Mojtaba Azimian, Azam Shahvarughi-Farahani, Mahdi Rahgozar, Masoud Etemadifar, Zahra Nasr

**Affiliations:** 1 Department of Clinical Sciences, School of Medicine, Tehran University of Social Welfare and Rehabilitation Sciences, Tehran, Iran; 2 Department of Neurology, School of Medicine, Isfahan University of Medical Sciences, Isfahan, Iran; 3 Department of Medical Sciences, School of Medicine, Medical Students’ Research Center, Isfahan University of Medical Sciences, Isfahan, Iran

**Keywords:** Depression, Fatigue, Iran, Multiple Sclerosis, Physical impairment

## Abstract

**Background:** Fatigue, depression, and physical impairment are common among multiple sclerosis (MS) patients. The aim of this study was to determine the relationship between depression, physical impairment, and fatigue in an Iranian MS cohort.

**Methods:** Fifty consecutive relapsing-remitting MS patients and 50 age- and sex-matched healthy controls (HCs) were recruited from Sina Rehabilitation Clinic, Tehran, Iran. The depression substance of Hospital Anxiety and Depression Scale (HADS), Expended Disability Status Scale, and Fatigue Severity Scale questionnaires were used to assess depression, physical impairment, and fatigue, respectively.

**Results:** This study included 38 (76%) females and 12 (24%) males in both patients and HC groups. The depression substance of the HADS in MS and HCs showed a mean value of 1.92 ± 0.80 and 1.17 ± 0.38, respectively (P < 0.001). Pearson’s correlation analyses showed that in the MS group depression was associated with fatigue (r = 0.54, P = 0.01), but not with physical impairment (r = 0.16, p = 0.01), while fatigue was associated with both depression (r = 0.54, P = 0.01) and physical impairment (r = 0.36, P = 0.01). Depression in HCs group was also associated with fatigue (r -0.64, P = 0.01).

**Conclusion:** Fatigue is associated with both depression and physical impairment, and an intervention in one of these conditions might improve others.

## Introduction

Depression is the most notable affective disorder associated with multiple sclerosis (MS), which has a negative impact on working ability, social relationships, and quality-of-life. Severely depressed MS patients are likely to be fatigued, and treating the depression may improve fatigue. However, the findings are controversial, several earlier studies either found no association between fatigue and depression, or only a modest association, while more recent studies support a substantial relationship.^1^^,^^2^ According to different studies, fatigue is reported by 75–95% of patients and many of them describe it as their most disabling symptom that limits their activity more than any others symptom.^3^ When compared with healthy controls (HCs), MS patients report more frequent and sever fatigue, which is aggravated by heat and physical effort and may worsen throughout the day.^4^ Numerous studies have attempted to unravel the association between fatigue and physical disability among patients with MS. While some of them found a positive relationship between physical impairment and fatigue, others could not reveal any relationship.^5^

This study is designed to determine the relationship between depression, fatigue, and physical impairment in an Iranian cohort of MS patients.

## Materials and Methods

Clinically define relapsing-remitting MS patients according to 2010 revised McDonald’s criteria were recruited from Sina Rehabilitation clinic in Tehran, Iran.^6^ The following criteria were used to select patients for inclusion in this cohort study:

- Age between 20 and 40 years old.
- Having a suitable mental status (Persian language validated Mini-Mental State Examination (MMSE) > 22).^7^
- Having an Expended Disability Status Scale (EDSS) score between 3.5 and 6.
- Not having any other mental or physical disease.
- Not having any relapse during the last 3 months before the assessment to be included in the study.

After the selection of the MS group, healthy sex- and age-matched adults from the relatives of MS patients were recruited as the control group. HCs also had a suitable mental status (MMSE > 22) and did not have any other mental or physical disease.

Fatigue was assessed using the Persian validated Fatigue Severity Scale (FSS).^8^ The FSS focused solely on physical symptoms of fatigue and is the most commonly used instrument to measure the severity of fatigue among individuals with MS. Depression was measured with the Persian validated depression substance of Hospital Anxiety and Depression Scale (HADS).^9^ The HADS contains 14 items and consists of two subscales: anxiety and depression. In this study, we used only depression substance.

The FSS and HADS were used as a self-reported tool, however, for patients who had difficulty in reading, a structured interview was carried out. EDSS was measured by an experience neurologist on a clinical examination.

Local ethical approval was granted and all patients signed an informed consent before inclusion in the study. Data analyses were carried out using Statistical Package for the Social Sciences (SPSS, version 20). Scores for all measures were tested for deviation from the normal distribution by means of the Kolmogorov-Sminov test. Patients and HCs were compared by using independent samples t-test. For both group a linear correlation analysis (Pearson’s coefficient) was conducted to estimate the relationship between fatigue and depression, physical impairment. Level of significance was set at P < 0.05.

## Results

The MS group had 38 (76%) females and 12 (24%) males and the HC group had 38 (76%) female and 12 (24%) males. MS patients had a mean age of 30.38 ± 6.15 years, a mean EDSS score of 4.02 ± 0.74 and a mean disease duration of 5.56 ± 3.03 years. HCs had a mean age of 30.38 ± 6.15 years, which was not statistically different from that of MS patients (P > 0.05).

Table 1 shows the comparisons between the mean fatigue and depression scores between the groups. Fatigue and depression scores were significantly higher in MS patients compared with HCs (P < 0.0001).

Pearson’s correlation analyses showed that in the MS group depression was associated with fatigue (r = 0.54, P = 0.01), but not with physical impairment (r = 0.16, P = 0.01), while fatigue was associated with both depression (r = 0.54, P = 0.01) and physical impairment (r = 0.36, P = 0.01). Depression in HCs group was also associated with fatigue (r -0.64, P = 0.01).

## Discussion

In this study, we investigated the relation between depression, physical impairment, and fatigue in MS in an Iranian MS cohort. According to our results, fatigue is related to depression and physical impairment, but there is no statistically significant correlation between physical impairment and depression. Furthermore, among the HCs, there is a positive correlation between fatigue and depression as well.

Multiple sclerosis related fatigue can be distinguished from fatigue associated with depression. First, MS related fatigue usually lasts for only a few hours in contrast to the more persistent fatigue associated with depression. Second, its aggravation by heat is unique for MS related fatigue. Third, the feelings attributed to depression such as hopelessness, sadness and anxiety, lack in MS fatigue.^1^

The underlying mechanism for the relationship between fatigue and depression remains unsolved. Fatigue in MS may occur as a symptom of the disease process itself and may be further amplified by the experience of fatigue during depressive episodes. Alternatively, depression may occur as a psychological reaction to the limitations on daily life caused by fatigue.^10^

**Table 1:** Depression and fatigue scores of MS patients (n = 50) and HC (n = 50)

	**HCs**	**MS**	**P**	**T**
HADS	1.17 ± 0.38	1.92 ± 0.80	0.000	−5.77
FSS	3.23 ± 1.08	5.03 ± 1.70	0.000	−6.58

FSS: Fatigue Severity Scale; HADS: Hospital Anxiety Depression Scale; HCs: Health controls; MS: Multiple sclerosis

However, the cross-sectional design of this study does not allow for drawing conclusions about the direction of causality between depression and fatigue.

While there is still no medication to fight fatigue, depression is a treatable condition. Therefore, by treating depression in MS patients, their fatigue might be improved. Physical impairment in our study was associated with fatigue. Fatigue could result in physical impairment and also physical impairment could result in fatigue by the fact that MS patients may require more energy to perform daily tasks and thus resulting in lack of energy and fatigue.

## Conclusion

Fatigue is associated with both depression and physical impairment, and an intervention in one of these conditions might improve others. However, the direction of such association is still not elucidative and further investigations needs to warrant this fact.
